# Study of the interaction effect of traumatic brain injury and functional polymorphisms of interleukin genes on the severity of schizophrenia

**DOI:** 10.1192/j.eurpsy.2026.11270

**Published:** 2026-07-31

**Authors:** V. E. Golimbet, T. V. Lezheiko, M. V. Gabaeva, N. Y. Kolesina, S. S. Koroleva

**Affiliations:** Clinical Genetics Laboratory, Mental Health Research Center, Moscow, Russian Federation

## Abstract

**Introduction:**

According to the neuroinflammation hypothesis, schizophrenia occurs as a result of environmental influences that cause an immune response. It is known that traumatic brain injury (TBI), which is considered an environmental risk factor for schizophrenia, is accompanied by neuroinflammatory processes, but its effect on the severity of schizophrenia has been poorly studied.

**Objectives:**

To investigate the relationship between TBI and the severity of symptoms in patients with schizophrenia, taking into account the modulating effect of polymorphisms of the genes of pro- and anti-inflammatory interleukins.

**Methods:**

The study included 1157 patients with schizophrenia (ICD-10 items F20, F21, F23.1, F25.). 452 patients had a history of TBI, 538 patients denied TBI. The severity of schizophrenia symptoms was measured using the PANSS. Three polymorphisms located in the promoters of the genes encoding interleukins IL-4(C-589T) rs2243250 and IL-10(C-592A) rs1800872 were selected for the genetic study.

**Results:**

A mutual effect of TBI and the interleukin gene variants IL-4(C-589T) rs2243250 and IL-10(C-592A) rs1800872 on the severity of schizophrenia symptoms was found. In particular, in the group of CC IL-4(C-589T) genotype carriers, the severity of negative and general psychopathological symptoms was higher than in carriers of the T allele. The IL-10(C-592A) rs1800872 polymorphism modified the effect of TBI on general psychopathological, but not positive or negative symptoms of schizophrenia. The severity of these symptoms was higher in carriers of the CC genotype compared to carriers of the A allele. In patients without TBI, the polymorphisms did not have a significant effect on the phenotype.

**Conclusions:**

Thus, our study has shown for the first time that the presence of a history of TBI and the patient’s genetic characteristics negatively affect the severity of schizophrenia.

**Disclosure of Interest:**

None Declared

